# Establishment and application of autoverification system for HbA1c testing

**DOI:** 10.11613/BM.2024.030705

**Published:** 2024-10-15

**Authors:** Ran Gao, Fang Zhao, Liangyu Xia, Chaochao Ma, Yingying Hu, Zhihong Qi, Xinqi Cheng, Ling Qiu

**Affiliations:** Department of Laboratory Medicine, Peking Union Medical College Hospital, Chinese Academic Medical Science and Peking Union Medical College, Beijing, China

**Keywords:** HbA1c, autoverification, reference change value, laboratory information system

## Abstract

**Introduction:**

This study aimed to determine autoverification rules for routine glycated hemoglobin (HbA1c) analysis based on high-performance liquid chromatography (HPLC) principle. Laboratory information system (LIS) and Bio-Rad D-100 Advisor software (Bio-Rad, Hercules, USA) with graphics recognition function were carriers for the autoverification system.

**Materials and methods:**

A total of 105,126 HbA1c results, including 98,249 HbA1c matching fast plasma glucose (FPG) results of real-world data from May 2019 to June 2020, were collected to determine autoverification rules including flags, delta checks, reporting limits, and logical rules. The validation database was composed of 48,045 HbA1c results and 41,083 matching FPG results. Autoverification passing rate and the reduction of turnaround time (TAT) were evaluated.

**Results:**

Four autoverification systems (A, B, C, D) were established by two types of delta check rules, 28 flags, one reporting limits, and two kinds of logical rules. The autoverification passing rates were 80.6%, 78.8%, 83.7%, and 81.3%, and the average time saved in TAT were 117.5 min, 116.7 min, 121.1 min, and 121.7 min, respectively.

**Conclusions:**

Autoverification system C was the optimal one. Application of distribution of FPG corresponding to HbA1c groups had better performance as logical rules. Established HbA1c autoverifcation system shortened the auditing report time and improved work efficiency.

## Graphical abstract

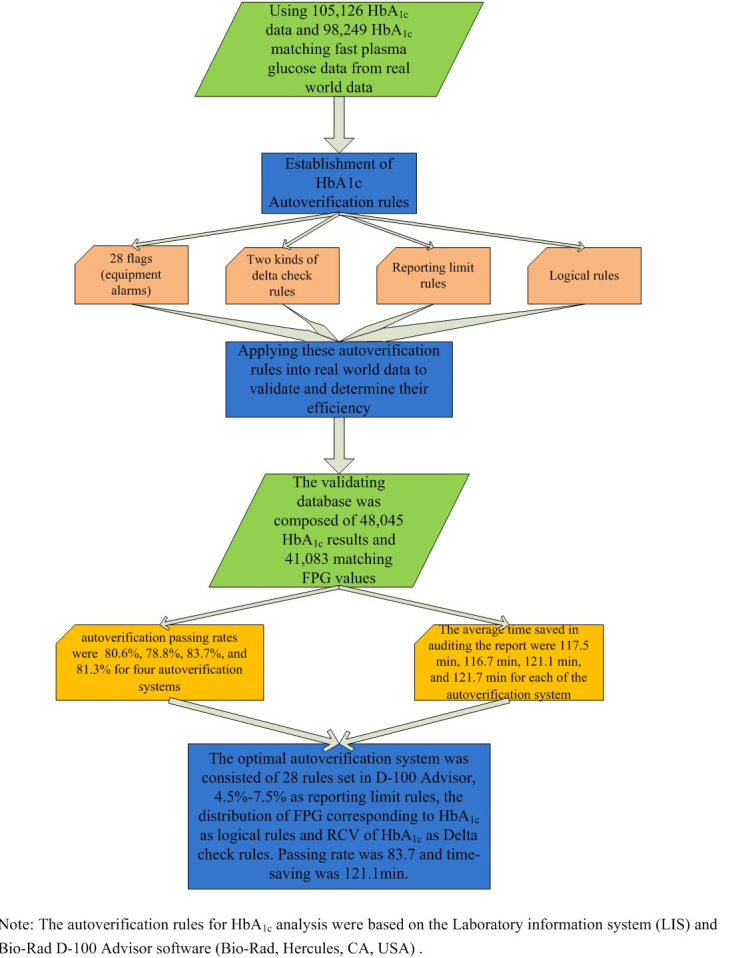

## Highlights

• Analysis of the chromatography map of HbA1c was involved in the autoverification procedure

•Real world data was used to establish autoverification rules for HbA1c

• The optimal auto-verification system of HbA1c results was set up based on the fast plasma glucose distribution according to HbA1c groups and reference change value

## Introduction

Reviewing of specimen test report is an important process in clinical laboratories. Manual verification is performed in most clinical laboratories to detect possible errors before the results are released, which is time consuming. Compared with the shortcomings of traditional manual verification, such as slow speed, uneven professional level of auditors, untimely discovery of abnormal results, and inevitable human errors, autoverification has the advantages of rapidity and objectivity. Previous studies have demonstrated that autoverification could shorten turnaround time (TAT), reduce labor requirement, minimize error rate, and allow clinical laboratory technologists to devote more attention to results with greater potential error (*1*, *2*). By reducing manual reviewing errors and TAT, autoverification plays an important role in improving medical safety.

Glycated hemoglobin HbA1c reflects plasma glucose concentrations over 2-3 months and is useful for monitoring glycemic control in patients with diabetes. In 2010, the American diabetes Association (ADA) recommended HbA1c as a diagnostic test for diabetes and prediabetes (*3*). Verification of HbA1c results is complex especially when HbA1c was detected by ion-exchange high-performance liquid chromatography (IE-HPLC), which involves chromatogram identification, such as checking whether the peak area is within the normal range and the HbA1c peak fits, appearance of unknown or abnormal peaks, with or without trailing, and baseline drift. Factors such as the logical relationship between HbA1c and blood glucose and/or glycosylated albumin concentrations can affect the correct reporting and interpretation of HbA1c results. Because of the complexity of the audit process of HbA1c results, there are currently no published data on the autoverification of HbA1c.

In this study, referred to the CLSI AUTO-10A international guidelines, we explored ways to set up rules for the autoverification of HPLC-determined HbA_1c_ to reduce artificial audit error and shorten TAT time (*4*).

## Materials and methods

### Subjects

This study was conducted in the clinical laboratory of the Peking Union Medical College Hospital. A total of 105,126 HbA1c results, including 98,249 fasting plasma glucose (FPG) matching results from outpatients and inpatients from May 2019 to June 2020, were used to establish autoverification rules. The validation database was composed of 48,045 HbA1c and 41,083 matching FPG results. All of the HbA1c results during these periods were included in the study, and no exclusion strategy was applied. Clinical diagnosis, age and sex information corresponding to each HbA1c result were collected simultaneously.

This study was approved by the Ethics Committee of Peking Union Medical College & Chinese Academy of Medical Sciences, Peking Union Medical College Hospital (ethical approval document number: S-K1007).

### Methods

Ethylenediaminetetraacetic acid-K2 (EDTA-K2)-containing tubes were used for HbA1c testing, which were measured using Bio-Rad D-100 (Bio-Rad, Hercules, USA) hemoglobin testing system based on ion-exchange high-performance liquid chromatography (IE-HPLC) principle. Altogether, 49,998 FPG results were analyzed on Roche Cobas C702 (Roche Diagnostics GmbH, Mannheim, Germany) coupled with the corresponding reagents and calibrators, and 48,251 FPG results were analyzed on Beckman Coulter AU5800 (Beckman Coulter Inc., Brea, USA) coupled with the corresponding reagents and calibrators for establishing autoverification rules. For validation 17,084 and 23,999 FPG results were collected each from Roche C702 and Beckman Coulter AU5800. Both of the analyzers use hexokinase method to detect glucose. We performed comparative study on the two instruments twice a year to ensure that FPG results were comparable. Twenty samples with FPG results ranged from 3.9 mmol/L to 30 mmol/L were collected for comparative study. The acceptance bias of the FPG comparative study was 7.0%, which was equal to the total allowable error (TEa) of glucose external quality assessment published by National Center for Clinical Laboratories in China (*5*).

Laboratory information system (LIS) was provided by Mediinfo (Zhejiang, China). The autoverification rules were converted into computer languages for integration into LIS.

#### Intermediate software and review rules

There are 32 preset HbA1c audit rules in the D-100 Advisor (Bio-Rad, Hercules, USA). Through analyzing alarms information in the past years and according to the actual needs of the laboratory, a total of 28 preset rules were adopted as HbA1c automatic audit rules, including the rules for determining the state of instruments, state of specimens, quality control checks, critical results and scope, and suspected variants. Explanation and proportion of alarms for each rule were given as Supplementary Table S1 in Appendix. If HbA1c result triggers one of these rules, the result will be flagged and a comment text will be sent to LIS. Four alarms that were ruled out were S window present, C window present, E window present and D window present, which had duplicated meaning and function with alarms ahead.

#### Reporting limits

Reporting limits were used to determine results that require verification other than analytical and critical values. HbA1c reference interval in our laboratory was 4.5-6.3%, and analytical range was 3.5-20.0% which was cited from Bio-Rad D-100 HbA1c Advisor Handbook, version 1.1. We analyzed the distribution of HbA1c results in all patients from May 2019 to June 2020 to help establish reporting limits of HbA1c. The 2.5th, 5th, 10th, 90th, 95th, and 97.5th percentiles of patient results distribution were calculated.

#### Logical rules

We designed and validated two types of logical rules: First rule was based on diabetes diagnostic criteria, which was HbA1c ≥ 6.5% and FPG ≥ 7.0 mmol/L and HbA1c < 6.5% and FPG < 7.0 mmol/L (*6*). Fasting plasma glucose concentration of 7.0 mmol/L is the diagnostic criteria for diabetes. Glucose concentration of 11.1 mmol/L was cut-off value of random plasma concentration for diagnosing diabetes. Cut-off value of impaired glucose tolerance (IGT) was 7.8 mmol/L. Second rule was based on the correlation between HbA1c and FPG (*7*-*9*). We grouped HbA1c results into nine groups with 1% as group spacing and calculated the distribution of FPG in each HbA1c group. The bottom group was HbA1c ≤ 5%, and the top group was HbA1c > 12%. In each HbA1c group, if the matched FPG results were within the mean ± 2 standard deviation (SD) among normally distributed data or 2.5th-97.5th percentiles among non-normally distributed data, HbA1c results would be considered fulfilling logical rules and marked pass. If logical rules weren’t fulfilled, results would go to manual review.

#### Delta check

We set six months as the upper limit of the delta check time interval and calculated the difference between the present HbA1c results and the most recent historical HbA1c results. Two kinds of delta check rules were set. One was the reference change value (RCV) of HbA1c, and another was absolute difference 0.5%. The calculation formula of RCV is as follows:



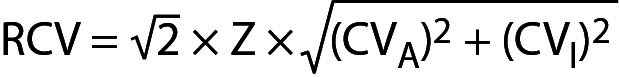



where CV_A_ is analytical coefficient of variation, CV_I_ within-subject biological variation and Z coverage factor (*10*).

HbA1c internal quality control (IQC) analysis was performed once a day. The coefficient of variation (CV) of IQC within one year was 2%, which was used as the CV_A_. We obtained CV_I_ of HbA1c from the European Federation of Clinical Chemistry and Laboratory Medicine website and that was 1.2%. We used RCV of the 99% confidence level, and the coverage factor Z was 2.58.

#### Set up of the autoverification rules

Two kinds of delta check rules and two types of logical rules were used to set up four autoverification systems, named A, B, C, and D:

A: flags + delta check RCV + reporting limit + logical rule 1
B: flags + delta check 0.5% + reporting limit + logical rule 1
C: flags + delta check RCV + reporting limit + logical rule 2
D: flags + delta check 0.5% + reporting limit + logical rule 2

The passing rate and report validation saving times of the four autoverification schemes were calculated, respectively. We set the time point at which the HbA1c results were released from D-100 as the starting point, while the time point of completely reviewing results as the end point. Between starting point and end point was the time consumed by reviewing. We analyzed the time required for reviewing when autoverification was not applied in validating database. As they were non-normally distributed, medians were present. When autoverification was applied, time for reviewing HbA1c results that passed autoverification would be less than 1min. Thus, the saving times could be figured out.

### Statistical analysis

Data management and statistical analyses were performed using Microsoft Excel 2010 (Microsoft, Redmond, United States) and SPSS statistical software (version 17.0; SPSS Inc., Chicago, USA). One-Sample Kolmogorov-Smirnov test was used to describe the distributions of HbA1c and FPG results. Normally distributed data are represented as mean ± SD, whereas non-normally distributed data are represented by median and 2.5^th^ and 97.5^th^ percentiles.

## Results

### Autoverification rules in the D-100 Advisor intermediate software

From May 2019 to June 2020, there were 387 flags sent from the HbA_1c_ analyzer to the LIS. Among them, 84.4% of the flags indicated suspicious hemoglobin variants, 11.4% indicated critical results, and 4.2% had specimen problems.

### Reporting limits

The HbA1c results at the 2.5th, 5th, 10th, 90th, 95th, and 97.5th percentiles were 4.8%, 4.9%, 5.0%, 7.5%, 8.4%, and 9.4%, respectively. Patients’ median HbA1c was 5.6% (5.3-6.3%). Through discussing with endocrinology specialists, 7.5% was set as the upper limit of HbA1c results. The lower limit of HbA1c reference interval, 4.5%, was set as the lower limit of reporting limits. Thus, the reporting limits was set as 4.5-7.5%.

### Logical rules

#### Logical rule 1

In the establishing database, 13.2% patients’ HbA1c results failed to pass this logical rule. As for the logical rule HbA1c < 6.5% and FPG < 7.0 mmol/L, HbA1c results less than 6.5% but with corresponding FPG concentrations higher than 7.0 mmol/L were failed to pass the logical rules. Likewise, for logical rule HbA1c ≥ 6.5% and FPG ≥ 7.0 mmol/L, HbA1c results higher than 6.5% but with corresponding FPG concentrations less than 7.0 mmol/L were rejected. We further explored the FPG concentrations of the rejected HbA1c results by subgrouping. The distribution of FPG in the rejected results is summarized in Table 1.

**Table 1:** The distribution of fasting plasma glucose of specimens failed to pass logical rules based on diabetes diagnostic criteria in establishing database

	**HbA1c results failed the rules**			
**Logical rules used for autoverification**	**HbA1c**	**Corresponding FPG distribution (mmol/L)**	**N (%)**	N* (%)
	6.1% (5.9-6.3)	7.0 ≤ FPG < 7.8	4731 (65.1)	
HbA1c < 6.5% and FPG < 7.0 mmol/L	6.1% (5.9-6.3)	7.8 ≤ FPG < 11.1	2478 (34.1)	7270 (7.4)
	6.1% (5.8-6.3)	FPG ≥ 11.1	61 (0.8)	
	6.9% (6.6-7.3)	6.1 ≤ FPG < 7.0	3347 (59.2)	
HbA1c ≥ 6.5% and FPG > 7.0 mmol/L	6.9% (6.6-7.5)	3.9 ≤ FPG < 6.1	2217 (39.2)	5654 (5.8)
	7.3% (6.8-8.3)	FPG < 3.9	90 (1.6)	
N - the number of HbA1c results that failed to pass logical rules in each FPG category. N*- the number of HbA1c results that failed to pass logical rules. FPG - fasting plasma glucose. HbA1c - glycated hemoglobin.				

#### Logical rule 2

In the total nine subgroups of HbA1c, the average rejection rate was 4.1%. Fasting plasma glucose distribution for each HbA1c subgroup and rejection rate were shown in Table 2.

**Table 2:** The proportion of patients that fail to pass the setup rules based on distribution of fasting plasma glucose corresponding to each HbA1c group in verification system

**HbA1c categories**	**N**	**Corresponding FPG (mmol/L)**	**Failed proportion %**
HbA1c ≤ 5%	5781	5.0 ± 1.2	2.2
5% < HbA1c ≤ 6%	59,002	5.3 ± 1.3	3.7
6% < HbA1c ≤ 7%	15,822	6.8 ± 2.8	3.7
7% < HbA1c ≤ 8%	6797	8.4 ± 4.2	4.1
8% < HbA1c ≤ 9%	3297	9.9 ± 5.8	3.8
9% < HbA1c ≤ 10%	1608	5.2-18.2	4.7
10% < HbA1c ≤ 11%	845	5.2-20.4	4.7
11% < HbA1c ≤ 12%	402	13.9 ± 8.4	5.5
HbA1c > 12%	248	5.6-26.0	4.4
HbA1c categories: HbA1c results were grouped with 1% as group spacing. N - the number of HbA1c results in each category. Corresponding FPG results are expressed as mean ± 2SD or 2.5th-97.5th percentile depending on data distribution. Failed proportion %: patients whose FPG results were not within the corresponding categories. FPG - fasting plasma glucose. HbA1c – glycated hemoglobin. SD - standard deviation.			

### Delta check

According to the calculation formula of RCV, the RCV at 99% confidence level for HbA1c in our laboratory was 8.5%. If the HbA1c delta percent change exceeded 8.5%, the present result would fail to pass delta check rule and ended with manual review. In validating database, RCV of HbA1c (8.5%) and the absolute delta difference of 0.5% were validated separately. The passing rates of this step were 98.8% and 95.4%, respectively. There were 1618 patients whose absolute HbA1c delta differences exceeded 0.5%, but the delta percent changes were within 8.5%.

### Validating results

**Figure 1:**
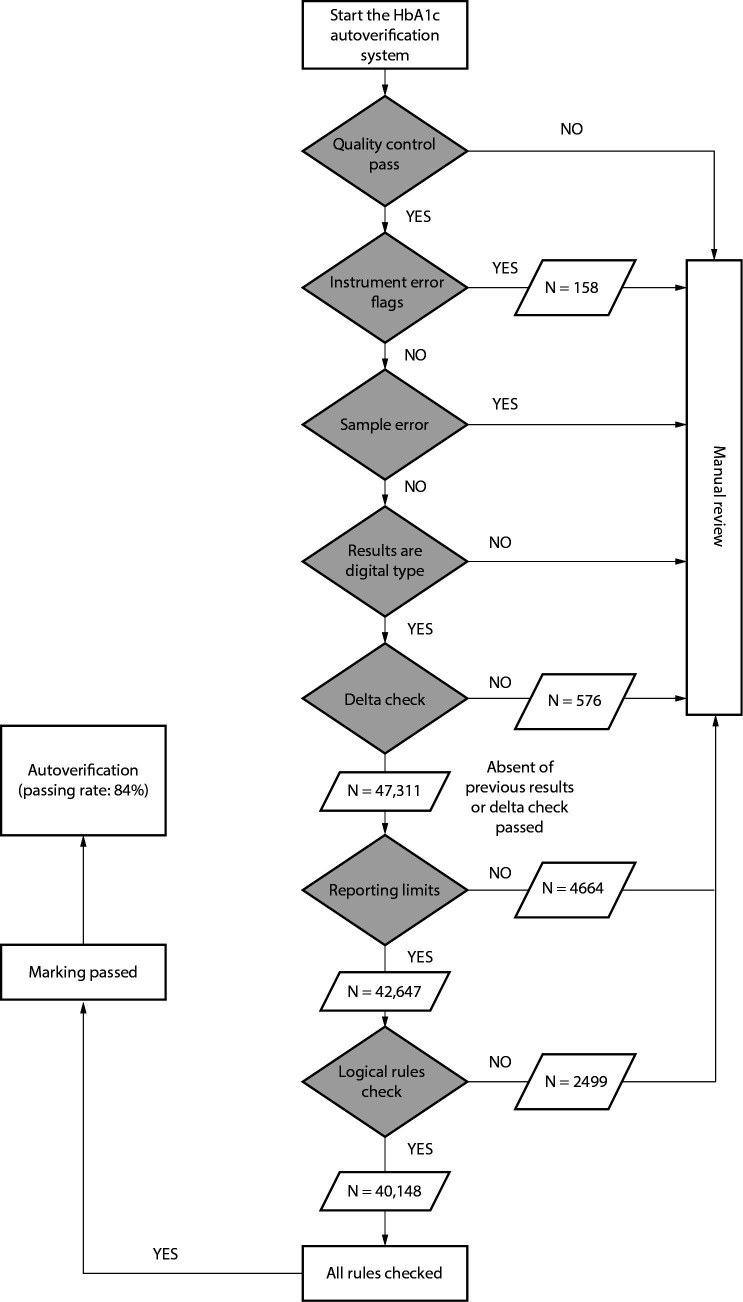
Flowchart of Autoverification system C for HbA1c characterized by reference change value as delta check and fasting plasma glucose distribution as logical rules

The passing rates of the four autoverification systems were 80.6% (A), 78.8% (B), 83.7% (C), and 81.3% (D), as shown in Table 3. Experienced operators evaluated the samples that failed to pass the autoverification system C. Flagged by D-100 Advisor was 0.33% of results, 1.09% results failed to pass delta check, 0.87% results ruled out by reporting limits rule, and 14.15% results were rejected by logical rule. These samples triggered at least one of the established rules and were correctly identified by using autoverification system. Autoverification flowchart of C system was shown in Figure 1.

**Table 3:** Validation of Autoverification systems of HbA1c on Bio-Rad D100 platform with Bio-Rad D100 Advisor and Laboratory Information System

**Autoverification systems**	**Validating samples (N)**	Passing samples (N*)	**Passing rate (%)**	**Time-saving (min)**
A	47,887	38,719	80.6	117.5
B	47,887	37,840	78.3	116.7
C	47,887	40,148	83.7	121.1
D	47,887	39,080	81.3	121.7
A - flags + delta check RCV 8.5% + reporting limit + logical rule 1. B - flags + delta check 0.5% + reporting limit + logical rule 1. C - flags + delta check RCV 8.5% + reporting limit + logical rule 2. D - flags + delta check 0.5% + reporting limit + logical rule 2. Logical rule 1: HbA1c < 6.5% and FPG < 7.0 mmol/L, HbA1c ≥ 6.5% and FPG > 7.0 mmol/L. Logical rule 2: logical rules based on the distribution of fasting plasma glucose corresponding to each HbA1c group. N - the number of samples involved in the validation. N* - the number of samples that successfully passed the autoverification review. Passing rate % - the proportion of samples that successfully passed the autoverification review. FPG - fasting plasma glucose. HbA1c - glycated hemoglobin. RCV - reference change value.				

The report validation saving times of the four autoverification systems were 117.5 min (A), 116.7 min (B), 121.1 min (C), and 121.7 min (D), respectively. The validation results are presented in Table 3.

## Discussion

As a diagnostic and monitoring indicator for diabetes mellitus, HbA1c is widely used in clinical course. In order to shorten TAT and reduce manual auditing error, this study explored autoverification system for HbA1c. The optimal system, autoverification system C, consisted of 28 rules set in D-100 Advisor, 4.5%-7.5% as reporting limit rules, the distribution of FPG corresponding to HbA1c as logical rules and RCV of HbA1c as delta check rules. The application of HbA1c autoverification system greatly shortened TAT.

We explored two approaches for delta check, one was RCV for HbA1c and another was absolute difference of 0.5%. Through validation of each delta check rule, we found that there was quite a few of patients with HbA1c delta differences exceeding 0.5%, while their delta percent changes were within 8.5%. Among these patients, 82.3% of them had diabetes or prediabetes and 7.2% had cancer, renal failure, or chronic renal dysfunction. Diabetes patients without proper glycemic control and type 1 diabetes patients would have larger fluctuations in HbA1c concentrations than other patients. In patients with cancer, especially those undergoing chemotherapy and radiotherapy or those with renal diseases, the synthesis of HbA1c would be affected, and the fluctuation might be different compared to other patients (*11*-*14*). Given that our hospital was a comprehensive hospital and HbA1c specimens came from various clinical departments, we chose RCV for HbA1c as delta check as it could effectively filter out patients with changes in condition.

In verification process we experimented two kinds of logical rules. The results showed that there were 13.2% patients with FPG or HbA1c elevated alone, which would cause a large number of HbA1c results that should not be intercepted flowing into manual review area by using diabetes diagnostic criteria as logical rules. This phenomenon indicated that the relationship between FPG and HbA1c was not optimistic, which was consistent with previous studies (*8*, *15*). One of the studies analyzed the relationship between HbA1c and plasma glucose (PG) concentrations at multiple time points. It showed that PG at prebreakfast time point had the weakest relationship with HbA1c (*8*). A cross-sectional study with 14,294 Chinese subjects verified the correlations between HbA1c and FPG. The results showed that the correlations were lower in HbA1c < 6.5% and FPG < 7.0mmol/L group compared with HbA1c ≥ 6.5% and FPG > 7.0mmol/L group (Pearson’s correlation coefficient: 0.34, 0.77, respectively) (*15*). Considering the instability and variability of the correlations between FPG and HbA1c and the diversity of patients and diabetes therapy, using diabetes diagnostic criteria as logical rules was unreasonable. Instead, the FPG distribution corresponding to each HbA1c group in our laboratory was more suitable as logical rules.

In the present study, we did not arbitrarily apply the reference interval (4.5-6.3%) of HbA1c as reporting limits. HbA1c results were recruited from various patients including physical examination population, undiagnosed patients with diabetes and patients undergoing diabetes treatment, which indicated that a significant proportion of patients had abnormal HbA1c results. Thus, the distribution of HbA1c results in our laboratory provided solid basis for establishing reporting limits. In addition, HbA1c concentration of 7.0-7.5% is recommended for older adults, while those with multiple coexisting chronic diseases, cognitive impairment, or functional dependence should have less stringent glycemic goals, such as HbA1c < 8.0-8.5% (*16*, *17*). The median age of the patients in our established database was 51 years (range, 38-62 years), and they were under diverse health conditions. More narrow reporting limits would give unnecessary burden on manual review, while wider reporting limits would fail to screen out HbA1c results that needed additional attention. For the sake of caution, we chose 7.5%, the 90^th^ percentile of HbA1c in our laboratory, as the upper limit for HbA1c autoverification.

Considering the abovementioned factors, autoverification system C is the most reasonable one. Validation results also suggest that autoverification system C had a higher pass rate than the other three autoverification systems.

Most of the flags sent by D-100 Advisor indicated the existence of hemoglobin variants. Hemoglobin variants are one of the main interfering factors in HbA1c testing, which underlines the importance of identifying abnormal peaks in the development of HbA1c autoverification. Our study combined graphical analysis with other traditional autoverification rules to form HbA1c auverification system. The combination could identify abnormal peaks efficiently and accurately, which not only reduced the time spending on manually screening but also improved medical safety.

Because detection of HbA1c is influenced by several conditions including method-specific and non-method-specific interferences, scientific literature on HbA1c autoverification is limited. Nonetheless, there are some studies on autoverificaion in other fields. Frameworks of the reported autoverification systems are similar, while main differences are the approaches to set autoverification rules. Reference interval and critical values are commonly used methods for establishing reporting limits rules (*2*, *17*). Calculating the distribution of previous results is another approach for setting reporting limits (*18*). Even though most of the autoverification system had absolute change and percentage change as delta check rules, using RCV as delta check rules has received more attention (*19*). We validated RCV and traditional delta check rules in our study, and found that the former is more powerful than the latter. Logical rules are the most difficult part in the whole system. Except few analytes have clear logical relationship, like total protein and albumin, the consistency between most analytes were not completely definite. Scientific literatures can give clues and directions on establishing logical rules. Chromatographic graphical analysis is very important for HbA1c auditing. This study provided a reference for laboratories that use HPLC or capillary electrophoresis methods to detect HbA1c. For other analytes, methods of setting reporting limits and delta check rules in our study can be considered. However, some limitations should be noted. First, 84.4% of error flags sent by HbA1c analyzer were indicated hemoglobin variants. Though the National Glycohemoglobin Standardization Program (NGSP) website states that Bio-Rad D-100 A1c program method will not be influenced by HbC, HbE, HbD and HbS, we still intercept specimens with error flags of hemoglobin variants and submit them to manual review. We will take further study on whether HbA1c results will be reliable with the presence of hemoglobin variants. Another limitation was that we did not include the red blood cell parameters in the logical relation with HbA1c due to the limited capacity of the information system. In the future, we will improve LIS capacity and integrate red blood parameter information with LIS to optimize HbA1c autoverification rules.

In conclusion, our study provided novel approaches, such as RCV of HbA1c and FPG distributions corresponding to HbA1c groups, to establish autoverification rules. HbA1c autoverification system significantly improved the speed of report verification and shortens the TAT.

## Data Availability

The data generated and analyzed in the presented study are available from the corresponding author on request.

## Appendix

**Table S1:** Autoverification rules in D-100 Advisor (Bio-Rad, Hercules, USA) intermediate software and the detection rate

	**Rules**	**Category**	**Explanation**	**Cut-off**	**Flag proportion (%)**
1	Total Area Low		The total area is less than the cut-off	50,000	2.1
2	Total Area High		The total area is higher than the cut-off	350,000	2.1
3	Unread Barcode	State of specimens	A sample tube or microvial barcode was not read	NA	0
4	No HbA1c		No HbA1c peak was identified	NA	0
5	No HbA0		No HbA0 peak was identified	NA	0
6	HbA1c Range	Critical results	The HbA1c result is outside the reportable range	3.5-20.0%	7.0
7	HbA1c High		The HbA1c result is greater than the cut-off	15%	4.4
8	E and S Present		Peaks are present in the E-Window and S-Window	NA	42.6
9	E and C Present		Peaks are present in the E-Window and C-Window	NA	9
10	D and S Present		Peaks are present in the D-Window and S-Window	NA	0.8
11	D and C Present		Peaks are present in the D-Window and C-Window	NA	2.8
12	S and C Present		Peaks are present in the S-Window and C-Window	NA	0
13	E and D Present		Peaks are present in the E-Window and D-Window	NA	0
14	Custom F		The F area is within a suspect range	5-30%	0.3
15	Minor Peaks > 10%	Suspected hemoglobin variant	The unknown 1-9, A_1a_, A_1b_, or P3 area is greater than the cut-off	10%	8.5
16	LA1c Cutoff		The LA1c area is greater than the cut-off	7%	0.5
17	HbS Cutoff		The S-Window area is greater than the cut-off	50%	0
18	HbC Cutoff		The C-Window area is greater than the cut-off	50%	0
19	HbD Cutoff		The D-Window area is greater than the cut-off	43%	1.3
20	HbE Cutoff		The E-Window area is greater than the cut-off	39.1%	18.6
21	HbF Cutoff		The F area is greater than the cutoff	30%	0
22	Baseline Slope		The A1c Slope-To-Area Ratio is outside the acceptable range	0.00-0.10	0
23	A1c Sigma		The A1c sigma is outside the acceptable range	0.30-0.92	0
24	A1c Tau	State of instrument	The A1c Tau is outside the acceptable range	0.168-1.500	0
25	A1c Fit Crest Time		The A1c Fit Crest Time Diff is outside the acceptable range	1.40-3.20	0
26	A0 Sigma		The A0 Sigma is outside the acceptable range	0.15-0.45	0
27	A0 Tau		The A0 Tau is outside the acceptable range	0.17-0.75	0
28	Pressure overshoot or undershoot		The pressure overshoot or undershoot is greater than the cut-off	7.5	0
HbA1c - glycated hemoglobin.					
